# Identification of a novel resistance (E40F) and compensatory (K43E) substitution in HIV-1 reverse transcriptase

**DOI:** 10.1186/1742-4690-5-20

**Published:** 2008-02-13

**Authors:** Marleen CDG Huigen, Petronella M van Ham, Loek de Graaf, Ron M Kagan, Charles AB Boucher, Monique Nijhuis

**Affiliations:** 1 Department of Medical Microbiology, University Medical Center Utrecht, The Netherlands; 2 Department of Infectious Diseases, Quest Diagnostics Incorporated, 33608 Ortega Hwy, San Juan Capistrano, CA 92690, USA

## Abstract

**Background:**

HIV-1 nucleoside reverse transcriptase inhibitors (NRTIs) have been used in the clinic for over twenty years. Interestingly, the complete resistance pattern to this class has not been fully elucidated. Novel mutations in RT appearing during treatment failure are still being identified. To unravel the role of two of these newly identified changes, E40F and K43E, we investigated their effect on viral drug susceptibility and replicative capacity.

**Results:**

A large database (Quest Diagnostics database) was analysed to determine the associations of the E40F and K43E changes with known resistance mutations. Both amino acid changes are strongly associated with the well known NRTI-resistance mutations M41L, L210W and T215Y. In addition, a strong positive association between these changes themselves was observed. A panel of recombinant viruses was generated by site-directed mutagenesis and phenotypically analysed. To determine the effect on replication capacity, competition and *in vitro *evolution experiments were performed. Introduction of E40F results in an increase in Zidovudine resistance ranging from nine to fourteen fold depending on the RT background and at the same time confers a decrease in viral replication capacity. The K43E change does not decrease the susceptibility to Zidovudine but increases viral replication capacity, when combined with E40F, demonstrating a compensatory role for this codon change.

**Conclusion:**

In conclusion, we have identified a novel resistance (E40F) and compensatory (K43E) change in HIV-1 RT. Further research is indicated to analyse the clinical importance of these changes.

## Background

Shortly after the introduction of Zidovudine (AZT) in 1987 it became clear that HIV-1 is able to develop resistance to this drug [1,2]. Now, after twenty years of NRTI usage in the clinic the complete pattern of resistance is still not understood. Multiple studies have identified mutations at (at least) six codons in the reverse transcriptase (RT) enzyme (thymidine analogue associated mutations (TAMs); M41L, D67N, K70R, L210W, T215Y/F and K219Q/E) that can cause a decrease in Zidovudine susceptibility [3-7]. HIV-1 develops these TAMs by two distinct pathways: the TAM-1 pathway consisting of T215Y, M41L, L210W and sometimes D67N or the TAM-2 pathway including T215F, K70R, K219Q/E and D67N [8-10]. These substitutions cluster around the dNTP binding pocket and confer resistance by increasing the excision of the incorporated nucleoside analogue from the DNA chain by a pyrophosphorolysis-like mechanism [11,12].

Recently, multiple epidemiological studies have identified novel mutations in HIV-1 RT showing a strong association with NRTI-treatment. These mutations include the K20R, V35M, T39A, E40F, K43E/Q/N, A98G, K122E, G196E, E203K/D, H208Y, D218E, H221Y, K223E/Q and L228H/R changes [13-20]. Statistical methods have shown positive associations with NRTI-resistance for these substitutions. The appearance of a lysine to glutamic acid change at position 43 (K43E) is strongly associated with NRTI-treatment [20]. This mutation has an even higher association with NRTI treatment when compared to specific known drug-resistance mutations such as M41L, K219E and K65R (Stanford HIV Drug Resistance database). Yet, it is unknown why this mutation is being selected. The glutamic acid to phenylalanine change at codon 40 (E40F) is the result of as much as three transversions and is absent in the untreated population. Both changes are particularly interesting since they are located in close proximity of the known M41L drug resistance mutation.

Novel amino acid changes can be selected during (NRTI) treatment for several reasons. They can reduce susceptibility to particular drugs and/or they can act as compensatory mutations by improving the viral replication capacity (RC). Alternatively, they can appear as a result of escape from immunological pressure on wild type amino acids [21]. It is important to understand the role of each of these single mutations for the management of therapy-failing patients and new drug development.

In this study we have investigated which mechanisms explain the appearance of the E40F and K43E substitutions during NRTI-treatment by generating a panel of site directed mutants and analysing their replication capacity as well as their drug sensitivities.

We have demonstrated that the E40F change results in an increase in Zidovudine resistance and a decrease in RC. The K43E does not decrease Zidovudine susceptibility but increases RC, when combined with E40F, acting as a compensatory mutation.

## Results

### Association of the E40F and K43E changes with NRTI-treatment and resistance

To better understand the role of the E40F and K43E substitutions we analyzed the frequencies of these substitutions in the Quest Diagnostics reference laboratory database containing more than 160,000 (RT) sequences from patients across the United States (1/1/1999–12/31/2005). Forty percent of these samples showed no genotypic evidence of resistance, according to the Quest Diagnostics resistance algorithm [22].

The overall variability at codon 40 and 43 was 1.2% and 6.9% respectively (Table 1). Among all changes at position 40, two occurred frequently either as a mixture or as homogenous population; the aspartic acid (D) was observed with a relative frequency of 52% and the phenylalanine (F) with a relative frequency of 29% (Table 1). The presence of E40F was limited to samples that contained additional (RTI) resistance-associated mutations (0.6%; Odds ratio: 363; p < 0.0001) however the frequency of E40D was not significantly different in predicted ARV-resistant and ARV-sensitive samples. The most prevalent change at position 43 was the glutamic acid (E), appearing in 47% of all mutant codons (Table 1). Also the K43E change was found in 5.3% of samples with other resistance mutations but only 0.1% of samples with no predicted resistance (OR = 52, p < 0.0001). Likewise, K43Q (resistant virus: 3.3%; OR: 23, p < 0.0001) and K43N (resistant virus: 1.8%; OR: 11; p < 0.0001) were found predominantly in association with other resistance mutations.

**Table 1:** Amino acid variation at codons 40 and 43 in HIV-1 reverse transcriptase

Codon 40	Number	pct of mut	pct of total	Codon 43	Number	pct of mut	pct of total
	
D	732	38.0%	0.452%	E	4262	38.1%	2.631%
F	541	28.1%	0.334%	Q	2444	21.8%	1.509%
E/D	264	13.7%	0.163%	N	1516	13.5%	0.936%
K	54	2.8%	0.033%	K/E	836	7.5%	0.516%
K/E	53	2.8%	0.033%	K/Q	699	6.2%	0.432%
A	50	2.6%	0.031%	R	379	3.4%	0.234%
E/Q	33	1.7%	0.020%	K/N	333	3.0%	0.206%
E/G	27	1.4%	0.017%	K/R	285	2.5%	0.176%
E/A	27	1.4%	0.017%	E/Q	122	1.1%	0.075%
Q	21	1.1%	0.013%	K/D/N/E	41	0.4%	0.025%
V	21	1.1%	0.013%	A	37	0.3%	0.023%
V/F	10	0.5%	0.006%	T	36	0.3%	0.022%
S/F	10	0.5%	0.006%	K/H/N/Q	34	0.3%	0.021%
<10 examples	81	4.2%	0.050%	M	33	0.3%	0.020%
				K/T	20	0.2%	0.012%
Total	1924	100.0%	1.188%	S	19	0.2%	0.012%
				N/H	14	0.1%	0.009%
				K/A/T/E	10	0.1%	0.006%
				<10 examples	69	0.6%	0.043%
				
				Total	11189	100.0%	6.908%

In a total of 161974 sequences the amino acid variation at codons 40 and 43 in reverse transcriptase was determined. Shown are the percentages of the specified amino acid change of all mutations at that position (pct of mut) and in the total population (pct of total).

We investigated the association of the E40F and K43E changes with each other and with the known thymidine associated mutations (M41L, D67N, K70R, L210W, T215Y/F and K219Q/E; Table 2). HIV-1 strains harbouring E40F and/or K43E showed the strongest association with all TAM-1 pathway mutations (M41L, L210W and T215Y). Mutations from the TAM-2 pathway (D67N, K70R, T215F and K219Q/E) were only weakly or even negatively associated with the E40F and K43E changes, with the exception of the D67N substitution, which has also been associated with the TAM-1 pathway. Interestingly, the E40F substitution change showed the highest association with K43E (OR of 38.2 and phi-value of 0.287, p < 0.0001).

**Table 2:** Association of 40F and 43E with thymidine analogue-associated mutations

pos1	pos2	phi	OR	% pos2 in pos1	P value
40F	43E	0.287	38.2	84%	<10E-09
40F	41L	0.125	6.9	99%	<10E-09
40F	210W	0.162	11.2	97%	<10E-09
40F	215Y	0.124	7.2	94%	<10E-09
40F	67N	0.107	6.6	79%	<10E-09
40F	70R	0.001	1.1	9%	*NS*
40F	215F	-0.001	0.9	4%	*NS*
40F	219E	0.032	4.6	14%	<10E-09
40F	219Q	-0.003	0.8	4%	*NS*

43E	40F	0.287	38.2	10%	<10E-09
43E	41L	0.326	6.3	91%	<10E-09
43E	210W	0.367	9.0	78%	<10E-09
43E	215Y	0.31	6.4	82%	<10E-09
43E	67N	0.211	4.8	58%	<10E-09
43E	70R	-0.002	0.9	9%	*NS*
43E	215F	0.014	1.4	7%	9.7E-08
43E	219E	0.009	1.4	4%	*NS*
43E	219Q	0.004	1.1	6%	*NS*

Binomial correlation coefficients (phi) and Odds Ratios (OR) were calculated for 57 amino acid substitutions at 34 reverse transcriptase codons to study the association of E40F and K43E with each other and with known thymidine analogue associated mutations.

Phi: binomial correlation coefficient (1.0 = perfect pairwise correlation). OR: Odds Ratio – the observed frequency of the pair divided by the product of the individual mutation frequencies. P-value: chisquare probability was evaluated for significance at a Benjamini-Hochberg false discovery rate (FDR) of 0.01 for 1,566 multiple comparisons.

NS: Not significant at FDR 0.01.

Sequences with a phi value of 0.15 or greater, an odds ratio of > 2 and FDR of 0.01 were considered to be co-varying.

We also noted a positive association between K43E and amino acid changes E44A, V118I, H208Y, K219N/R and V75M (data not shown; p values were highly significant at an FDR level of 0.01 in all cases).

### Resistance to Zidovudine (AZT)

Both mutations are co-varying with TAM-1 pathway mutations and therefore we determined the effect of the E40F and K43E changes on thymidine analogue resistance (Zidovudine) in a set of clinically relevant reference viruses (Table 3). The introduction of the E40F change in the background of M41L and T215Y resulted in a 14-fold further increase in Zidovudine-resistance when compared to the M41L+T215Y double mutant. Introduction of the K43E change did not lead to a change in IC_50 _for Zidovudine in the viruses that were tested.

**Table 3:** Zidovudine susceptibility analysis

Resistance-associated amino acid in RT	40	41	43	184	210	215	219	Average			Fold
Consensus B	E	M	K	M	L	T	K	IC50 (nM)			Increase
Wild type (HXB2)	.	.	.	.	.	.	.	114	±	10	
Wild type+K43E	.	.	E	.	.	.	.	90	±	36	1×
M41L+T215Y	.	L	.	.	.	Y	.	1544	±	402	14×
M41L+T215Y+E40F	F	L	.	.	.	Y	.	21307	±	8810	187×
M41L+T215Y+K43E	.	L	E	.	.	Y	.	1556	±	496	14×
M41L+T215Y+E40F+K43E	F	L	E	.	.	Y	.	15350	±	5022	135×

Pat A	F	L	E	V	W	Y	T	14739	±	3105	129×
Pat A-WT40	.	L	E	V	W	Y	T	1596	±	377	14×
Pat A-WT43	F	L	.	V	W	Y	T	13127	±	4582	115×

Pat A-derived virus clone revealed additional amino acid changes including T200I, R211K, V245T, E248D, I293V and E297H

The mean IC_50 _values of at least two experiments are shown (± standard error of the mean); in the most right column the fold increase compared to the wild type HIV-1 HXB2 reference strain is shown.

Furthermore, a virus clone containing the N-terminal part of RT of a patient-derived virus isolate (Pat A) containing both E40F and K43E changes was made. This clone displayed high-level resistance to the thymidine analogue Zidovudine (129-fold increase in IC_50_) when compared to the wild type reference strain HXB2 (Table 3).

Changing codon 40 back to wild type in the patient A-derived virus clone (Pat A-WT40) resulted in a 9-fold decrease in IC_50 _for Zidovudine. This indicates that this single amino acid change is responsible for a 9-fold further increase in Zidovudine resistance in the highly resistant Pat A-derived virus clone (Table 3). In contrast, changing codon 43 back to wild type (Pat A-WT43) did not lead to a change in Zidovudine resistance.

### Effect of E40F change on RC

**Figure 1:**
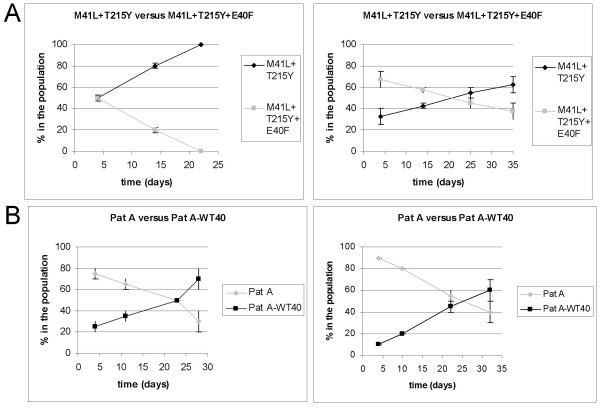
**Replication competition experiments with E40F site-directed mutants**. Replication competition experiments were performed in SupT1 cells in at least two independent experiments. After four days and after 2, 4 and 6 passages the relative presence of both viruses in the culture was determined by sequencing. Shown are two representative experiments. The variability in each independent experiment is indicated by ± standard error of the mean (SEM). A: M41L+T215Y versus M41L+T215Y+E40F B: Pat A (E40F, M41L, K43E, M184V, L210W, T215Y and K219T) versus Pat A-WT40 (M41L, K43E, M184V, L210W, T215Y and K219T).

We determined whether the E40F change causes resistance at the cost of reducing replicative capacity by performing competition experiments. Indeed, the introduction of the E40F change in the background of M41L and T215Y resulted in a gradual reduction of the M41L+T215Y+E40F, indicating that the E40F change results in a clear decrease in RC (Fig. 1A). Also, changing the mutation back to wild type at codon 40 in the patient-derived virus clone (Pat A-WT40) improved the RC of this virus (Fig. 1B).

### Effect of K43E change on RC

**Figure 2:**
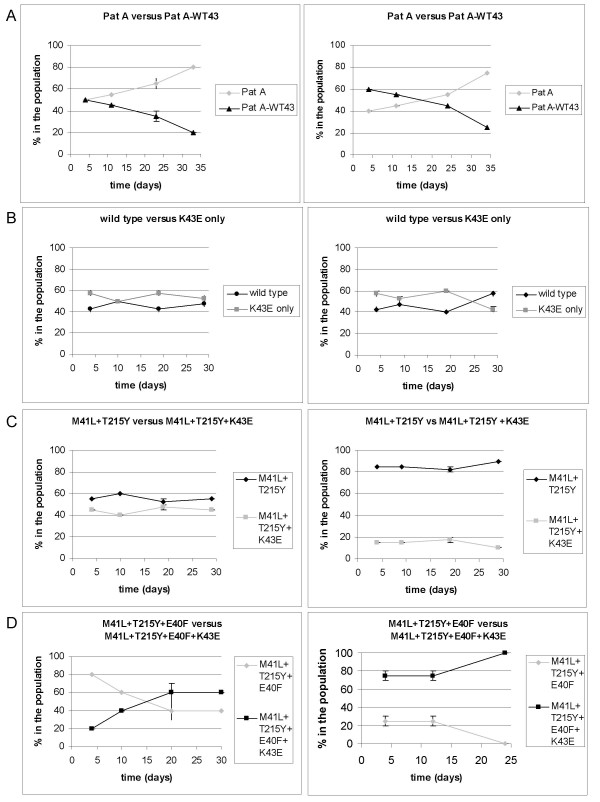
**Replication competition experiments with K43E site-directed mutants**. Replication competition experiments were performed in SupT1 cells in at least two independent experiments. After four days and after 2, 4 and 6 passages the relative presence of both viruses in the culture was determined by sequencing. Shown are two representative experiments. The variability in each independent experiment is indicated by ± standard error of the mean (SEM). A: Pat A (E40F, M41L, K43E, M184V, L210W, T215Y and K219T) versus Pat A-WT43 (E40F, M41L, M184V, L210W, T215Y and K219T). B: wild type versus wild type+K43E. C: M41L+T215Y versus M41L+T215Y+K43E. D: M41L+T215Y+E40F versus M41L+T215Y+E40F+K43E.

To determine if the K43E change has a compensatory role by increasing the viral RC, replication competition experiments were performed using a panel of site-directed mutants. Changing the mutant K43E codon to the wild type codon in the Pat A virus clone (Pat A-WT43) resulted in a reduction of viral RC (Fig. 2A), clearly indicating that the K43E change has a compensatory role in this patient-derived virus clone. In addition, we determined its effect in the wild type reference virus or the recombinant virus M41L+T215Y (Fig. 2B and 2C). These assays did not reveal any effect of the K43E change in the wild type or the M41L+T215Y background.

### Association between E40F and K43E

We hypothesized that the K43E substitution could be compensatory for the E40F substitution, since these changes are highly associated with each other (Table 2). The K43E change was found in 84% of all E40F-containing viruses. To determine if the K43E change is indeed compensatory for the deleterious effect of the E40F mutation on viral RC, the additional effect of K43E in the background of M41L+T215Y+E40F was determined. Indeed, replication competition experiments showed that the addition of K43E resulted in an increase in viral RC (Fig. 2D). Again, we found that the introduction of the K43E change in the M41L+T215Y+E40F virus did not lead to a significant change in Zidovudine resistance (Table 3), indicating that the effect of K43E (in the presence of E40F) is compensatory on the viral RC.

### In vitro evolution experiments

In vitro evolution experiments in the absence of drugs were performed for all virus clones (Table 4). In one out of four experiments the presence of the K43E change could lead to the acquisition of a change at position 215 (T-to-I, Table 4). This may indicate that the RC of this virus can be improved by a change at position 215 and may suggest an interplay between position 43 and 215 in RT.

**Table 4:** In vitro evolution experiments

		40	41	43	122	184	200	210	211	214	215	219	245	248	272	277	293	297
	HXB2	E	M	K	E	M	T	L	R	L	T	K	V	E	P	R	I	E
**M41L+T215Y**	**start**	**-**	**L**	**-**	**-**	**-**	**-**	**-**	**-**	**-**	**Y**	**-**	**-**	**-**	**-**	**-**	**-**	**-**
	experiment A	.	.	.	.	.	.	.	.	.	.	.	.	.	.	.	.	.
	experiment B	.	.	.	.	.	.	.	.	.	.	.	.	.	.	.	.	.
	experiment C	.	.	.	.	.	.	.	.	.	.	.	.	.	.	.	.	.
	experiment D	.	.	.	.	.	.	.	.	.	.	.	.	.	.	.	.	.
**M41L+T215Y+E40F**	**start**	**F**	**L**	**-**	**-**	**-**	**-**	**-**	**-**	**-**	**Y**	**-**	**-**	**-**	**-**	**-**	**-**	**-**
	experiment A	.	.	.	.	.	.	.	.	.	.	.	.	.	.	.	.	.
	experiment B	.	.	.	.	.	.	.	.	.	.	.	.	.	.	.	.	.
	experiment C	.	.	.	.	.	.	.	.	.	.	.	.	.	.	.	.	.
	experiment D	.	.	.	.	.	.	.	.	.		.	.	.	.	.	.	.
**Pat A**	**start**	**F**	**L**	**E**	**K**	**V**	**I**	**W**	**K**	**F**	**Y**	**T**	**T**	**D**	**A**	**K**	**V**	**H**
	experiment A	.	.	.	.	.	.	.	.	.	.	.	.	.	.	.	.	.
	experiment B	.	.	.	.	.	.	.	.	.	.	.	.	.	.	.	.	.
	experiment C	.	.	.	.	.	.	.	.	.	.	.	.	.	.	.	.	.
	experiment D	.	.	.	.	.	.	.	.	.	.	.	.	.	.	.	.	.
**Pat A-WT40**	**start**	**-**	**L**	**E**	**K**	**V**	**I**	**W**	**K**	**F**	**Y**	**T**	**T**	**D**	**A**	**K**	**V**	**H**
	experiment A	.	.	.	.	.	.	.	.	.	.	.	.	.	.	.	.	.
	experiment B	.	.	.	.	.	.	.	.	.	.	.	.	.	.	.	.	.
	experiment C	.	.	.	.	.	.	.	.	.	.	.	.	.	.	.	.	.
	experiment D	.	.	.	.	.	.	.	.	.	.	.	.	.	.	.	.	.
**K43E only**	**start**	**-**	**-**	**E**	**-**	**-**	**-**	**-**	**-**	**-**	**-**	**-**	**-**	**-**	**-**	**-**	**-**	**-**
	experiment A	.	.	.	.	.	.	.	.	.	T/I	.	.	.	.	.	.	.
	experiment B	.	.	.	.	.	.	.	.	.	.	.	.	.	.	.	.	.
	experiment C	.	.	.	.	.	.	.	.	.	.	.	.	.	.	.	.	.
	experiment D	.	.	.	.	.	.	.	.	.	.	.	.	.	.	.	.	.
**M41L+T215Y+K43E**	**start**	**-**	**L**	**E**	**-**	**-**	**-**	**-**	**-**	**-**	**Y**	**-**	**-**	**-**	**-**	**-**	**-**	**-**
	experiment A	.	.	.	.	.	.	.	.	.	.	.	.	.	.	.	.	.
	experiment B	.	.	.	.	.	.	.	.	.	.	.	.	.	.	.	.	.
	experiment C	.	.	.	.	.	.	.	.	.	.	.	.	.	.	.	.	.
	experiment D	.	.	.	.	.	.	.	.	.	.	.	.	.	.	.	.	.
**M41L+T215Y+ E40F+K43E**	**start**	**F**	**L**	**E**	**-**	**-**	**-**	**-**	**-**	**-**	**Y**	**-**	**-**	**-**	**-**	**-**	**-**	**-**
	experiment A	.	.	.	.	.	.	.	.	.	.	.	.	.	.	.	.	.
	experiment B	.	.	.	.	.	.	.	.	.	.	.	.	.	.	.	.	.
	experiment C	.	.	.	.	.	.	.	.	.	.	.	.	.	.	.	.	.
	experiment D	.	.	.	.	.	.	.	.	.	.	.	.	.	.	.	.	.
**Pat A-WT43**	**start**	**F**	**L**	**-**	**K**	**V**	**I**	**W**	**K**	**F**	**Y**	**T**	**T**	**D**	**A**	**K**	**V**	**H**
	experiment A	.	.	.	.	.	.	.	.	.	.	.	.	.	.	.	.	.
	experiment B	F/L	.	.	.	.	.	.	.	.	.	.	.	.	.	.	.	.
	experiment C	.	.	.	.	.	.	.	.	.	.	.	.	.	.	.	.	.
	experiment D	.	.	.	.	.	.	.	.	.	.	.	.	.	.	.	.	.

Shown are all amino acid changes present at start of the experiment compared to wild type (HXB2) and after 10 passages compared to baseline. All evolution experiments were performed in four independent experiments (A-D).

Introducing the wild type amino acid at codon 43 in the Pat A-virus clone (Pat A-WT43) could lead to a change of the E40F amino acid change (E40F/L) in one out of four experiments. This may indicate that in the absence of the positive effect of the K43E change on RC, the virus can improve its RC by removal of the E40F change.

## Discussion

In the present study the reasons for appearance of two novel changes at codon 40 and 43 in HIV-1 RT in patients failing nucleoside therapy were investigated. The E40F and K43E changes belong to a growing list of newly identified mutations that are associated with primary NRTI-resistance [13-20]. In this study we show that these E40F and K43E changes are highly associated with mutations from the TAM-1 pathway (M41L, L210W and T215Y) and less with the amino acid changes from the TAM-2 pathway (D67N, K70R, T215F and K219Q/E) (Table 2). This nicely confirms the previous described association of K43E, K43N and K43Q with some TAM-1 mutations [20]. Although mutations in the TAM-1 pathway have demonstrated to confer high-level NRTI-resistance, there also appears to be a selective pressure that allows generation and selection of these novel mutations. It could be possible that these mutations were overlooked in the past, but another, perhaps more plausible, explanation is the current widespread use of highly active antiretroviral therapy (HAART). Before 1995, four NRTIs (Zidovudine, Didanosine, Zalcitabine and Stavudine) were the only HIV-drugs approved for usage in the clinic and the well known TAMs were identified in this time period [3-7]. Hereafter Lamivudine, several non-nucleoside RTIs (NNRTIs) and protease inhibitors were approved by the Food and Drug Administration. 3TC and NNRTIs have been shown to select respectively for changes such as M184V and Y181C that resensitize TAM-containing HIV-1 RT to Zidovudine by decreasing the excision of this drug [12,23-26]. In agreement, Gonzales et al. have demonstrated that the frequency of K43E correlated with the number of previously received NRTI [15,20].

We hypothesize that the current HIV-treatment regimens force HIV to select for novel resistance patterns to further increase resistance. At the same time these resistant viruses may have a considerable loss in replicative capacity and could therefore select for additional changes that compensate the losses in RC.

To unravel the specific roles of the E40F and K43E we investigated their effect on drug susceptibility and replication by studying recombinant viral isolates as well as site directed mutants.

We have shown that the mechanisms explaining their appearance were different for both amino acid substitutions. Selection of the E40F change is driven by an increase in resistance to Zidovudine (nine to fourteen-fold). An increase in IC50 value was observed each time this change was introduced. This resistance effect appeared at the cost of a loss in RC for all combinations carrying this change.

In contrast, the appearance of the K43E change can be explained mainly by effects on the replicative capacity. This change appears to be a compensatory mutation that allows the resistant virus to increase its replicative capacity.

Compensatory mutations that increase viral replicative capacity without an effect on resistance have been described extensively for protease inhibitor resistance. Although a few studies have reported the compensatory effect of some mutations in RT, such as changes at codon 163, 74, 75, 63, 189, 230 and 396, this concept is relatively new for RTI resistance [27-32]. Considering the error-prone nature of HIV replication, a reverse transcriptase mutant will, similar to a PI-resistant virus, evolve towards a more fit virus in its environment if this is possible. Thus, although one could argue that for yet unknown reasons it may be more difficult for the virus to develop compensatory changes, it is more likely that compensatory changes in RT have not been sufficiently studied.

Considering the much lower prevalence of E40F compared to K43E, the selection of the latter mutation can not be explained solely in terms of compensation for the E40F change. This could imply that there are more changes in RT that cause a reduction in RC and as such could benefit from the appearance of the compensatory change at position 43. For instance, mutations at codons 44 and 118 associated with dual resistance to Zidovudine and 3TC are much more common than E40F [33-35]. The association of K43E with changes at these codons or H208Y, K219N/R and V75M changes may potentially involve a compensatory interaction, but further studies will be necessary to investigate this relationship.

Also, host (cellular) factors can be a reason for selection of amino acid changes in RT. Viral cytotoxic T lymphocyte (CTL) escape mutations that can be selected may prevent proteasomal cleavage, confer a decrease in transporter associated with antigen processing (TAP) transport efficiency, prevent MHC-I binding or lower CTL recognition. Several reports have shown that selection of the M41L change makes the epitope ALVEICTEM(EK) (amino acid 33 to 41/43) more immunogenic [36,37]. We hypothesized that the K43E change may (partially) compensate for this increased immunogenicity. Predictions using Netchop, a neural network based prediction method, suggested that addition of the K43E change in the background of M41L reduces the chance of proteasomal cleavage [38,39]. The resulting effect on CTL escape might positively influence the selection of the K43E change in patients with specific HLA-types. In conclusion, our results suggest that an RC-compensating mutation (K43E) could have an additional effect on CTL escape. Thus, we have to be aware of the interplay between the selection pressure of the immune system and viral replication capacity during (suboptimal) treatment. Further research is warranted to determine the influence of the immune system on the selection of novel mutations in RT.

The observation that the E40F and K43E substitutions are highly associated with TAM-1 changes, suggests that the biochemical mechanism of action is most likely an interaction with these changes. Modelling these mutations into the 3D structure of the RT catalytic complex did neither immediately suggest a structural basis for the mechanisms of resistance nor for any compensatory effects [40]. Although both mutations are located in close vicinity to the M41L residue, the structural basis for resistance to Zidovudine for the latter mutation itself is not obvious. This is because residue 41 is positioned ~8Å from the putative site for the ATP used in Zidovudine excision and thus cannot apparently directly influence ATP binding [41]. Rather M41L may have a more indirect effect on ATP binding perhaps via alteration of van der Waals contacts with F116, itself a site of a resistance mutation as well as being adjacent to the nucleotide interacting residue Y115. We speculate that the aromatic residue at codon 40 (F) could exert a similar indirect mechanism to affect excision of Zidovudine. However, further studies are warranted to determine the structural and biochemical explanation for their effects.

## Conclusion

We have identified a novel resistance (E40F) and compensatory (K43E) amino acid change in HIV-1 RT. Further studies are warranted to understand the mechanism of compensation. For clinical management it is important to be aware of novel resistance patterns such as the one conferred by the E40F that is currently not represented in the algorithms that are used to manage patients failing nucleoside therapies.

## Methods

### Analysis of E40F and K43E prevalence and associations

A dataset of 161,974 deidentified HIV-1 subtype B clinical samples sequenced at Quest Diagnostics Nichols Institute, San Juan Capistrano, CA from 1999 through 2005 was used to determine the prevalence of amino acid substitutions at reverse transcriptase codons 40 and 43.

A further more recent dataset of 139,443 samples collected as above between 2002 and June 2006 were used to analyze covariation between 40F, 40D, 43E and 43Q and 53 reverse transcriptase amino acid substitutions at 32 additional RT codons associated with resistance. Mixed amino acid calls were excluded from the analysis. Binomial correlation coefficients were calculated for 1,566 amino acid pairs. Chi square values were corrected for multiple comparisons with the Benjamini Hochberg correction (Benjamini and Hochberg 1995) with a false discovery rate (FDR) set to 0.01.

### Patient

Patient A was originally described by Nijhuis et al. as patient C0011 [42]. This patient was treated with Lamivudine (3TC) monotherapy and selected the M184V change. Subsequently, Zidovudine (AZT) was added to the regimen and a temporary decline in HIV-1 RNA levels was observed. Genotyping revealed that the increase in RNA load was associated with the appearance of M41L, L210W and T215Y. Later on, a further increase in HIV-1 RNA level was observed and the viral genotype showed the appearance of the E40F and K43E changes.

### Cells

MT2 cells and SupT1 cells were cultured in RPMI 1640 medium, supplemented with L-glutamine (Cambrex, Verviers, Belgium), 10% heat-inactivated foetal calf serum (FCS, Invitrogen) and 10 μg/ml gentamicin (Invitrogen) and passaged twice a week. 293T cells were cultured in Dulbecco's modified Eagle's medium (DMEM, Cambrex), supplemented with 10% FCS and 10 μg/ml gentamicin. All cells were maintained at 37°C and 5% CO_2_.

### Generation of recombinant virus clones

HIV-1 nucleic acids were isolated according to the method described by Boom *et al *and the N-terminal part of RT was amplified as previously described [43,44].

The amplified N-terminal part of RT was used to generate recombinant virus clones containing amino acid 25 to 314 from RT in a wild type (HXB2) backbone as described previously [44].

### Site-directed mutagenesis

To determine the influence of the E40F and/or K43E amino acid substitutions, these changes were introduced in a wild type reference strain (HIV-1 HXB2) and a virus clone harbouring the M41L and T215Y amino acid changes. Furthermore, these substitutions were changed to wild type in the patient A-derived recombinant virus clone. The N-terminal part of RT of the corresponding plasmid was amplified by Vent^® ^polymerase (New England Biolabs) with RT-BalI (5'ATG GCC CAA AAG TTA AAC AAT GG-3', nucleotides 2599–2621), RT-21 (5'-CTG TAT TTC TGC TAT TAA GTC TTT TGA TGG-3', nucleotides 3539–3510) and a third primer introducing the nucleotide change(s).

To introduce the E40F change in the M41L+215Y reference plasmid primers 40F-RT1 (5' GAA ATT TGT ACA **T**AG CTG GAA AAG G-3', nucleotides 2655–2679), 40F-RT2 5' GAA ATT TGT ACA **TT**G CTG GAA AAG G-3', nucleotides 2655–2679) and 40F-RT3new 5' GAA ATT TGT ACA **TTT **CTG GAA AAG GA-3', nucleotides 2655–2680) were used. To change the E40F substitution to wild type in the patient A-derived virus clone (Pat A-WT40) primer 40E-RT (5'-GAA ATT TGT ACA **GAG **TTG GAA GAG G-3', nucleotides 2655–2679) was used.

To introduce the K43E change in respectively the wild type plasmid, the plasmid containing M41L+T215Y or the M41L+T215Y+E40F plasmid, the primers HXB2-43E (5' ACA GAG ATG GAA **G**AG GAA GGG AAA A-3', nucleotides 2664–2688), 43E-RT (5' ACA GAG CTG GAA **G**AG GAA GGG AAA A-3', nucleotides 2664–2688) and 43E-RTA (5' ACA TTT CTG GAA **G**AG GAA GGG AA-3', nucleotides 2664–2686) were used. To delete the K43E substitution from the patient A-derived virus clone (Pat A-WT43), the corresponding plasmid was amplified with as third primer 43K-RT (5'-ACA TTT TTG GAA **A**AG GAA GGA AA-3', nucleotides 2664–2686).

Plasmid DNA was denatured for 2 minutes at 94°C, followed by 30 cycles of 30 seconds denaturation at 94°C, 30 seconds annealing at 55°C and 2 minutes extension at 72°C. The latter 20 cycles had an extension of 5 seconds for each elongation step and the amplicons were further elongated for 5 minutes at 72°C. Following genotypic analysis, virus clones were generated containing the desired amino acid change(s), while the remaining part of the genome was unchanged.

### Drug susceptibility analysis

The susceptibility for Zidovudine was determined using a cell-killing assay in MT2 cells, essentially as described before [45]. Phenotypic resistance was determined by measuring the fold increase in 50% inhibitory concentration (IC_50_) compared with the IC_50 _of the wild type HIV-1 HXB2 reference strain.

### Replication competition experiments

To determine the relative RC of several virus variants, competition experiments were performed in SupT1 cells by mixing two recombinant viruses based on TCID_50 _[46]. In a total volume of 1 ml, 2 × 10^6 ^SupT1 cells were infected at a total multiplicity of infection (m.o.i.) of 0.001. After 2 hours of infection at 5% CO_2 _and 37°C, cells were washed and subsequently cultured in 10 ml fresh culture medium. When full-blown syncytia were present in the culture ca. 50 μl virus supernatant was used to infect 2 × 10^6 ^new SupT1 cells until six passages were performed. RNA was extracted from culture supernatant at several time points during the experiment and the N-terminal part of RT was amplified and sequenced as described before. The relative presence of both variants in the population was determined by estimating the relative peak heights of the electropherograms. For each experiment a forward and reverse primer was analysed and the mean value (± SEM) is shown in the figure.

### *In vitro *evolution experiments

To determine the potential evolutionary pathways of the virus clones we performed *in vitro *evolution experiments. Therefore, 2 × 10^5 ^SupT1 cells were infected with 50 μl of a recombinant virus clone in 2 ml culture medium. The virus replication was monitored by determining the cytopathic effect. When most of the cells formed syncytia, the viral supernatant was harvested (10 minutes at 1800 g) and ca. 50–100 μl viral supernatant was used for a new passage. After ten passages, the N-terminal part of RT was sequenced as described before.

## Abbreviations

RT(I): reverse transcriptase (inhibitor)

RC: replication capacity

## Competing interests

The authors declare that they have no competing interests.

## Authors' contributions

MCDGH, PMH and LG performed the experiments. MCDGH, CABB and MN participated in the design of the study and writing the manuscript. RMK carried out the analyses of the associations with resistance. All authors read and approved the final manuscript.
